# The synergistic effect of a graphene nanoplate/Fe_3_O_4_@BaTiO_3_ hybrid and MWCNTs on enhancing broadband electromagnetic interference shielding performance

**DOI:** 10.1039/c7ra12909b

**Published:** 2018-01-08

**Authors:** Lun Jin, Xiaomin Zhao, Jianfeng Xu, Yanyu Luo, Danqing Chen, Guohua Chen

**Affiliations:** Polymer Science & Engineering Department, Huaqiao University Xiamen 361021 China hdcgh@hqu.edu.cn +86-592-6162280

## Abstract

In this work, methyl vinyl silicone rubber (VMQ) nanocomposites were prepared by solution blending VMQ, a graphene nanoplate/Fe_3_O_4_@BaTiO_3_ hybrid (GFBT) and MWCNTs, aiming to improve the electromagnetic interference (EMI) shielding performance of VMQ. Using the low defect graphene nanoplates (GNPs) as a carrier of Fe_3_O_4_@BaTiO_3_ nanoparticles, the GFBT hybrid was synthesized using a two-step solvothermal method. The micro morphology observed by scanning and transmission electron microscopy (SEM and TEM) showed that Fe_3_O_4_ (∼200 nm) and BaTiO_3_ (∼20 nm) were successfully loaded over GNPs. The GFBT hybrid and MWCNTs had good dispersion in the as-prepared VMQ/GFBT/MWCNTs (VGFBTM) nanocomposite. With a loading of 16.1 wt% total filler (GFBT : MWCNTs = 5 : 1), the shielding effectiveness (SE) of the VGFBTM composite ranged from 26.7 to 33.3 dB (>99.8% attenuation) in a wide frequency range of 1.0–20.0 GHz. A synergistic effect between the GFBT hybrid and MWCNTs provided good dielectric loss and magnetic loss, which played a significant role in improving the electromagnetic interference shielding effectiveness of VMQ. Besides, the electrical conductivity of the VGFBTM nanocomposite was improved compared with VMQ owing to the conducting network structure which was built from two-dimensional GNPs and one-dimensional MWCNTs.

## Abstract 2

In this work, methyl vinyl silicone rubber (VMQ) nanocomposites were prepared by solution blending VMQ, graphene nanoplate/Fe_3_O_4_@BaTiO_3_ hybrid (GFBT) and MWCNTs, aiming to improve the electromagnetic interference (EMI) shielding performance of VMQ.

## Graphical abstract

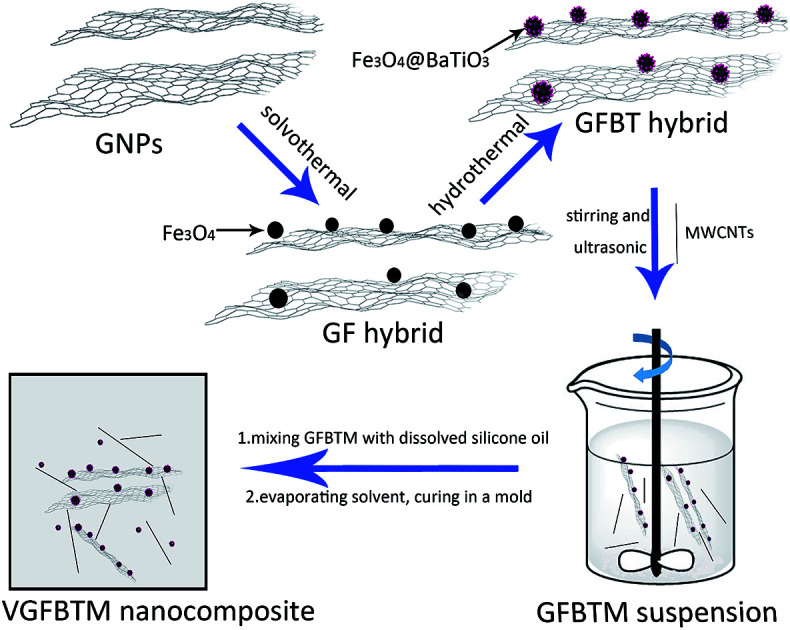

## 1. Introduction

In the rapidly evolving information age, the numerous applications of electronic products have created a convenient life for humans, whereas they have introduced serious electromagnetic interference (EMI) pollution^1,2^ as well. Electromagnetic waves generated by electronic equipment adversely affect other devices and living creatures, resulting in problems such as insufficient precision and dysfunction of the devices, or even being harmful to our health. Generally, electromagnetic interference shielding effectiveness (EMI SE) of a polymer composite depends mainly on the filler's intrinsic conductivity, dielectric constant, magnetic permeability and aspect ratio.^3,4^ In view of the above aspects, excellent EMI performance can be obtained when the filler has good intrinsic conductivity.^5,6^ The traditional EMI shielding or absorbing materials are single component such as carbon black, ferrite^7^ and graphite^8,9^ which have been studied in the past decade. In recent years, with electromagnetic pollution becoming more serious, researchers are focusing on graphene-based multiple nanocomposites to improve the EMI SE of polymers due to its super electronic conductivity and ability to act as a carrier of other absorbers such as RGO@MoS_2_,^10^ and RGO/SiO_2_/Fe_3_O_4_ hybrids.^11^ Currently, graphene mainly prepared by chemical oxidation reduction and mechanical stripping methods are two kinds of graphene based conductive fillers. In this work, GNPs has been used which possesses excellent electrical, mechanical, barrier and carrier properties due to their unique size and morphology during mechanical stripping process.

According to shielding mechanism, it is used to effectively enhance the EMI SE with the combination of carbon filler and other absorbers such as ferrimagnetic materials.^12–14^ Fe_3_O_4_ nanofiller has been widely studied as a promising absorber in polymer composites owning to high permeability and obvious absorbing loss to electromagnetic wave.^15^ However, the agglomeration and poor dispersion of these nanofillers in polymer matrices is the first problem to us. Without well dispersion in polymer composite, the composite can hardly perform ideal EMI SE. To solve the problem, various synthetic methods such as solvothermal method of graphene/Fe_3_O_4_ hybrids are reported and performed well EMI SE.^16,17^ Studied by microscopic characterization, Fe_3_O_4_ nanoparticles are anchored on the surface of graphene, meanwhile both of Fe_3_O_4_ nanoparticles and graphene show no more agglomeration.

The ideal EMI shielding composites not only require excellent magnetic permeability but also superior dielectric constant. The dielectric ceramic such as BaTiO_3_ shows obvious frequency dispersion characteristics and dielectric polarization effect which can lead to dielectric loss in the 2–18 GHz band.^18–21^ Here, the dielectric constant decreases with the increase of frequency, and the dielectric loss angle has extreme value.^22^ Guo *et al.*^18^ revealed that 15 wt% RGO@BaTiO_3_ in poly(vinylidene fluoride) matrix exhibited the highest value of reflection (−45.3 dB) and broad frequency bands (<−10 dB). All in all, it produces induced charge and weakens the electromagnetic field when surrounded by an extra electromagnetic field. Non-conductive absorbers such as Fe_3_O_4_ and BaTiO_3_ nanoparticles can decrease conductivity of graphene-based composites which is a problem we meet. Recently the combination of two-dimensional graphene with one-dimensional CNTs is used to build three-dimensional space conductive network which played a crucial role for high electrical conductivity in the composites.^23^

In this work, graphene nanoplates/Fe_3_O_4_@BaTiO_3_ hybrid (GFBT) was synthesized by loading Fe_3_O_4_ and BaTiO_3_ nanoparticles on graphene nanoplates *via* a two-step solvothermal method. MWCNTs was used as a synergist to weaken the negative effects of Fe_3_O_4_ and BaTiO_3_ nanoparticles on conductive property. The synergistic effect of GFBT hybrid and MWCNTs was studied on electromagnetic interference shielding property and electrical conductivity of methyl vinyl silicone rubber (VMQ). The micro morphology and structure of GFBT hybrid were characterized by SEM, TEM, X-ray powder diffraction (XRD) and Raman. The dispersion of GFBT hybrid and MWCNTs in VGFBTM nanocomposite was characterized by SEM. By optimizing the loading of GFBT and MWCNTs in VMQ, the effective EMI SE bandwidth with SE > 26.7 dB was over 1.0–20.0 GHz in a thickness of 2.6 mm and the electrical conductivity reached ∼0.01 S cm^−1^.

## 2. Experimental section

### 2.1 Chemicals and materials

Methyl vinyl silicone oil (COSIL® V-10000), hydrogenated silicone oil (COSIL® SH-80), platinum catalyst platinum catalyst (COSIL® CAT-Pt050) and inhibitor were supplied by Jiangsu Cosil of the new materials Co., Ltd., China. Graphene nanoplates (KNANO) and Multi-walled carbon nanotubes (TIME NANO® MTNM3; ∼98 wt% purity, OD = 10–20 nm and length = 10–30 μm; supplied by timenano, China) were dried in a vacuum oven at 80 °C for 24 h. Ferric chloride (FeCl_3_), polyethylene glycol (PEG, *M*_w_ = 1500), ethylene glycol, sodium acetate (CH_3_COONa), barium hydroxide octahydrate (Ba(OH)_2_·8H_2_O), titanium dioxide (TiO_2_), sodium dodecylbenzenesulfonate (SDBS), hexane were purchased from Xia'men chenhong Technology Co., Ltd. All these materials and chemicals were used as received without further purification. Deionized water was used in all experiments.

### 2.2 Experimental

#### 2.2.1 Synthesis of GNPs/Fe_3_O_4_ (GF) hybrid and GNPs/Fe_3_O_4_@BaTiO_3_ (GFBT) hybrid

0.39 g of GNPs and a certain amount of FeCl_3_ were dispersed into ethylene glycol by sonicating (2000 W, 20 KHz) for 6 min. CH_3_COONa and PEG were added into the suspension, followed by another 6 min ultrasonic. Then the mixture was poured into a Teflon-lined stainless-steel autoclave. After reacting at 200 °C for 10 h, a typical solvothermal process^24^ was finished. The black product GF hybrid was obtained by magnetic separation. Then, it was washed by deionized water and ethanol each for three times. Finally, the GF hybrid was dried in a vacuum oven overnight at 60 °C.

The GFBT hybrid were synthesised by hydrothermal method.^25^ 1.00 g as-prepared GF hybrid, a certain amounts of Ba(OH)_2_·8H_2_O and TiO_2_ were dispersed into 100 mL deionized water. The mixture was ultrasonicated for 6 min, then transferred to a Teflon-lined stainless-steel autoclave (150 mL). After heating at 200 °C for 10 h, the black product GFBT was separated by magnet and washed with deionized water for three times. The GFBT hybrid was dried in a vacuum oven overnight at 60 °C before used.

#### 2.2.2 Preparation of GFBT/MWCNTs (GFBTM) suspension

The GFBT hybrid, MWCNTs and SDBS were dispersed with a weight ratio of 10 : 2 : 1 in hexane under strong stirring and ultrasonic for 6 min to create a homogeneous suspension. The GFBTM suspension was prepared for later use.

#### 2.2.3 Preparation of VMQ/GFBTM (VGFBTM) nanocomposites

**Fig. 1:**
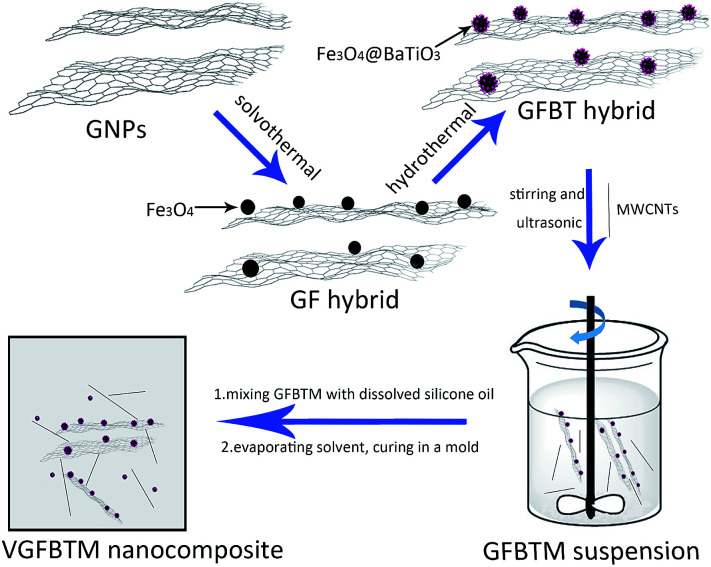
The synthesis process of the VGFBTM nanocomposites.

The VGFBTM nanocomposites were prepared *via* solvent blending method and curing process. The synthesis process was described in Fig. 1. First, Methyl vinyl silicone oil was dissolved in hexane under strong stirring and ultrasonic for 6 min, forming a homogeneous solution. Then the dissolved silicone oil and the GFBTM suspension were mixed under ultrasonic for 6 min. Then the mixture was slowly stirred overnight to completely evaporate the hexane solvent. Finally, the obtained mixture was cured with hydrogenated silicone oil in present of platinum catalyst and inhibitor at 80 °C for 2 h. In this work, the abbreviation of V1–V6 represented the six different samples of VGFBTM composites. For example, if the GFBTM filler content was 2.3 wt%, the name of the composite was V1 (Table 1).

**Table 1:** The specific parameters of components and the short name of corresponding composites in detail

Samples number	VMQ (g)	GFBT (g)	MWCNTs (g)	GFBTM (wt%)	Samples name
1	10	0.2	0.04	2.3	V1
2		0.4	0.08	4.6	V2
3		0.6	0.12	6.7	V3
4		0.8	0.16	8.8	V4
5		1.2	0.24	12.6	V5
6		1.6	0.32	16.1	V6

### 2.3 Characterization

The morphologies of samples were characterized by field emission scanning electron microscopy (FESEM, JSM-6700F) and transmission electron microscopy (TEM, JEM-2010 JEOL). Raman spectra was recorded with a He–Ne laser (532 nm) as the excitation source by Labram spectrometer (Super LabRam II system), and used to analyse GNPs, GF and GFBT hybrids. X-ray diffraction (XRD) patterns were recorded with a D8-Advance Instrument (Bruker AXS) using Cu Kα radiation generated at a voltage of 40 kV and a current of 40 mA. The range of 2*θ* was from 5 to 80 with a scanning rate of 5 per minute. The magnetic properties were measured on a NQTM-DC-001 vibration sample magnetometer (VSM) with a magnetic field of −20 000 to 20 000 Oe. The electrical conductivities (*σ*_v_) of the composite sheets were collected using Keithley 2400 source meter. Electromagnetic shielding were carried out using Agilent E8362B Vector Network Analyzer in 1.0–20.0 GHz microwave range. The round-shape VGFBTM samples with 2.60 mm thickness were placed inside the cavity of the sample holder which matches the internal dimensions of the 1–20 GHz wave guide. All the measurements were operated at room temperature.

## 3. Results and discussion

### 3.1 Microstructure and morphology

**Fig. 2:**
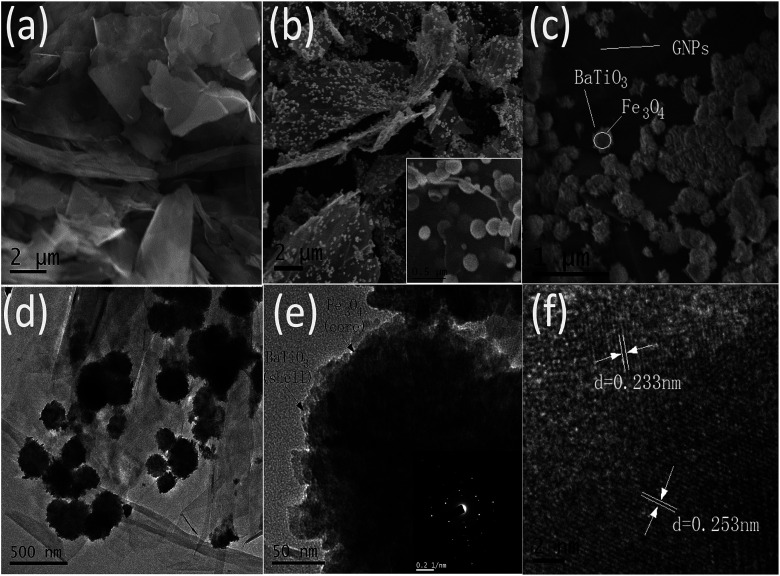
SEM images of (a) GNPs, (b) GF and (c) GFBT hybrid; (d) TEM images of GFBT hybrid and (e, f) HRTEM images of FBT particle.

The surface morphologies and sizes of GNPs, GF and GFBT hybrid were investigated using SEM and TEM. Typical SEM images of GNPs, GF and GFBT hybrid showed in Fig. 2a–c. As seen in Fig. 2a, the commercial GNPs obtained by mechanical stripping method presented lamellar structures with distinct wrinkled surface. Fig. 2b showed the SEM image of GF hybrid prepared by solvothermal method and the insert image of Fig. 2b showed the Fe_3_O_4_ nanospheres with an average diameter of 200 nm. The Fe_3_O_4_ nanospheres uniformly anchored on the surface of GNPs, which enlarged the layer space of graphene sheets and prevented restacking of GNPs as well. Fig. 2c–d showed the SEM and TEM micrographs of GFBT hybrid. Large quantities of BaTiO_3_ nanoparticles (∼20 nm) were coated on Fe_3_O_4_ nanospheres. It could be clearly seen in Fig. 2c that the surface of Fe_3_O_4_ nanospheres was no longer smooth and rounded, but wrapped in a thin and rough layer of BaTiO_3_ nanoparticles. In Fig. 2e, TEM image of Fe_3_O_4_@BaTiO_3_ (FBT) nanoparticles showed that light-colored BaTiO_3_ (shell structure) were coated on dark-colored Fe_3_O_4_ (core structure). The insert image in Fig. 2e was the corresponding selected area electron diffraction (SAED) pattern of GFBT hybrid, which demonstrated the monocrystalline structure of Fe_3_O_4_ and BaTiO_3_ nanoparticles. From Fig. 2f, HRTEM image of FBT nanoparticles indicated that the lattice plane spacing of the Fe_3_O_4_ particles was 0.253 nm (311) and the lattice plane spacing of the BaTiO_3_ particles was 0.233 nm corresponding to the (111) plane of BaTiO_3_ phase. It could further reveal that the synthesised Fe_3_O_4_ and BaTiO_3_ nanoparticles were monocrystallines.

**Fig. 3:**
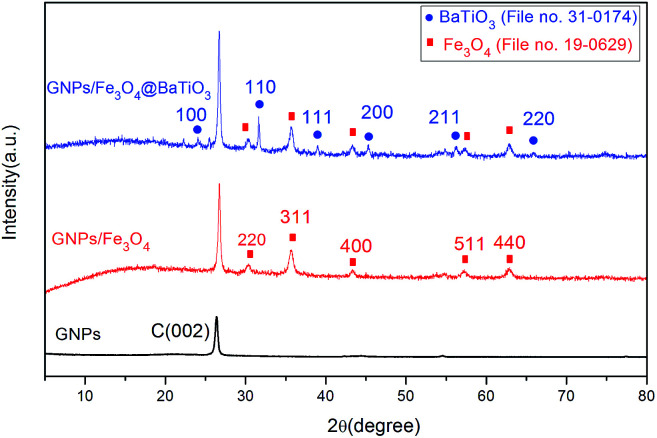
XRD patterns of GNPs, GF and GFBT hybrids.

The phase and structures of GNPs, GF and GFBT hybrid were studied by XRD. Fig. 3 showed the XRD patterns of GNPs, GF and GFBT hybrid. For GNPs, a conventional stacking peak of GNPs appeared around 2*θ* = 26°, indicating GNPs stack together easily and form graphitic structures. As to GF hybrid, the detected diffraction peaks of Fe_3_O_4_, (220), (311), (400), (511) and (440) were assigned to the face centered cubic structure of Fe_3_O_4_ (JCPDS card, file no. 19-0629). An additional intense diffraction peak around 26° corresponding to C (002) indicates that Fe_3_O_4_ formed on GNPs successfully. Compared with the above results, the XRD pattern of GFBT showed more characteristic diffraction peaks, (100), (110), (111), (200), (211) and (220) were assigned to the pure tetragonal perovskite structure of BaTiO_3_ (JCPDS card, file no. 31-0174). Besides, two-step solvothermal process did not affect Fe_3_O_4_ crystalline structure. The XRD results showed that Fe_3_O_4_ and BaTiO_3_ were successfully formed on GNPs after two-step solvothermal process.

**Fig. 4:**
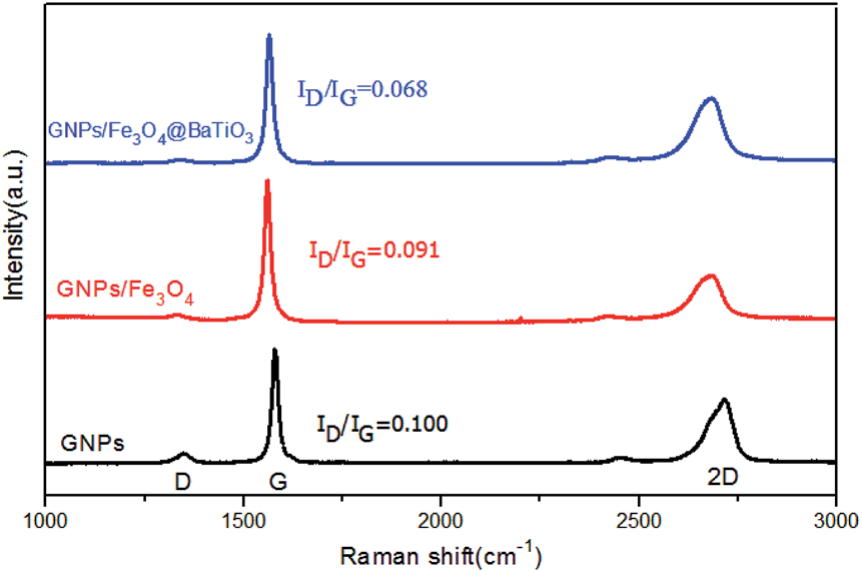
Raman spectra of GNPs, GF and GFBT hybrid.

Raman spectroscopy is a common and efficient method for the characterization of graphene materials. Herein, Raman spectra of GNPs, GF and GFBT hybrids performed in the 1000–3000 cm^−1^ range were presented in Fig. 4. Raman spectra of GNPs exhibited three regular peaks that the D-band line was around 1347 cm^−1^, G-band line was around 1578 cm^−1^ and 2D-band line was around 2716 cm^−1^. Here, the D band corresponds to the defect of graphene, which reflects the disorder of the graphene sheet. The G band corresponds to the first-order scattering of the E_2g_ mode observed for sp^2^ carbon domains, while the 2D peak in graphene is due to two phonons with opposite momentum in the highest optical branch.^26,27^ The intensity ratio of D and G (*I*_D_/*I*_G_) provides an effective index for comparing the lattice defects and the graphitization degree of carbon materials. The intensity ratio of 2D and G (*I*_2D_/*I*_G_) provides an index for identifying layers of graphene-based materials.^28,29^ As seen in Fig. 4, the *I*_2D_/*I*_G_ of GNPs was less than 1, meaning the graphene consisted of multiple layers. The *I*_D_/*I*_G_ of GNPs was 0.100, which meant few lattice defect in the sheet and edge of graphene. The *I*_D_/*I*_G_ (0.068) of GFBT hybrid was the lowest intensity in the three samples, indicating the oxygen-containing groups and lattice defect of GNPs decreased after the two-step solvothermal reaction process.

### 3.2 Magnetic properties

**Fig. 5:**
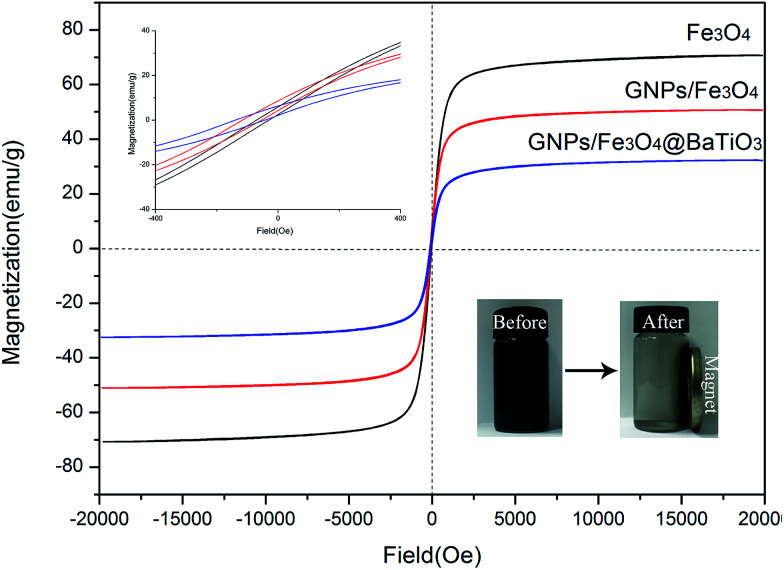
Hysteresis loops of Fe_3_O_4_, GF and GFBT hybrids at room temperature. The insets are the magnified views of the hysteresis loops at low applied fields and a digital of GFBT hybrid separated from the aqueous suspension by a magnet.

The magnetic properties of Fe_3_O_4_, GF and GFBT hybrids were measured by VSM at room temperature. The hysteresis loops curves of each sample was described in Fig. 5. The specific magnetic parameters including saturation magnetization (Ms), coercivity (Hc), and remanent magnetization (Mr) were listed in Table 2. In Fig. 5, compared to Fe_3_O_4_ nanoparticle, GF and GFBT hybrids exhibited the lower Ms values owing to the nonmagnetic properties of GNPs and BaTiO_3_. Nevertheless, effectively magnetic separation was still shown when the magnet was close to a GFBT suspension (as shown in right corner insert images in Fig. 5). The experiment indicated that GFBT hybrid had paramagnetic feature as well. From Table 2, Hc and Mr of GFBT hybrid were 150.0 Oe and 8.0 emu g^−1^, respectively. The two low values further verified the superparamagnetic feature of GFBT hybrid. In addition, the low Hc of GFBT hybrid led to the low resonance frequency, which meant a considerable magnetic loss to electromagnetic wave.^30–32^

**Table 2:** Magnetic parameters of Fe_3_O_4_, GF and GFBT hybrid

Samples	Parameters		
	Ms (emu g^−1^)	Hc (Oe)	Mr (emu g^−1^)
Fe_3_O_4_	70.7	71.4	6.1
GF	50.6	114.1	8.0
GFBT	32.3	150.0	6.5

### 3.3 Microstructures of VGFBTM composites

**Fig. 6:**
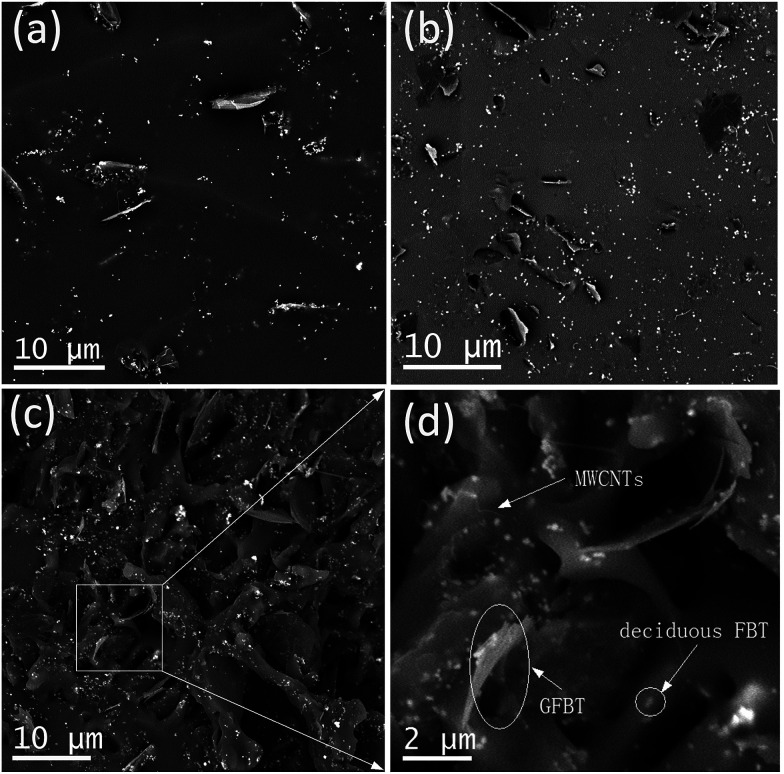
SEM images of (a) V1, (b) V4, (c) and (d) V6 composites.

In VQM composites, the dispersion status of the nanofillers played critical roles in determining the final properties of the composites. Fig. 6a–d showed SEM images of the V1, V4 and V6 composites. In the VGFBTM composites, GFBT hybrid and the deciduous FBT nanoparticles were randomly distributed on the fractured surface of the composites. The FBT nanoparticles anchoring on graphene sheets prevented stacking of the graphene sheets in the VGFBTM composites. In the case of V1 composite (Fig. 6a), graphene sheets were separated far from each other in the matrix. With the increasing content of GFBTM filler, the spatial network structures was gradually formed in Fig. 6c–d. Numerous small pore structures and spaces would be favorable for the attenuation of electromagnetic wave by absorption once the microwave gets into the nearly closed spaces.

### 3.4 Electrical conductivities of VGFBTM composites

**Fig. 7:**
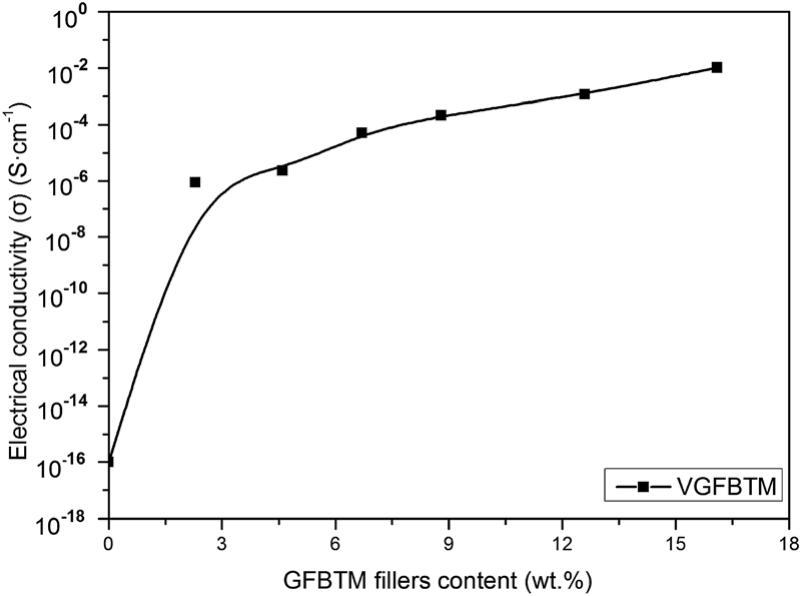
Plots of electrical conductivity *vs.* GFBTM fillers content for VGFBTM composites.

Fig. 7 showed variation in the electrical conductivity of VGFBTM composites with increasing GFBTM fillers (GFBT : MWCNTs = 5 : 1) content. The electrically insulating Fe_3_O_4_ and BaTiO_3_ nanoparticles attached on GNPs sheets may negatively affect electrical conductivity of the composites. Theoretically, GNPs have high electrical conductivity in the in-plane direction and MWCNTs have high electrical conductivity in the axis direction both owing to the sp^2^ hybrid. It means that they have high electrical resistance in other directions. Herein, one-dimensional MWCNTs acted as a bridge to connect two-dimensional GNPs layers and provided additional channels for the electron transfer within the VMQ matrix.^33^ Compared to the pure VMQ sheet, the VGFBTM composites showed an obvious increase in conductivity, mainly attributing to a decrease in the contact resistance as well as the formation of an efficient percolating network which was formed by GNPs and MWCNTs in VMQ matrix.^34^ The V6 composite showed good electrical conductivity which reached 0.01 S cm^−1^ with a 16.1 wt% total filler loading. The observations of electrical properties were confirmed from the SEM micrographs of the VGFBTM composites in Fig. 6, indicating spatial network structure is advantageous for conductivity.

### 3.5 EMI shielding efficiencies of VGFBTM composites

**Fig. 8:**
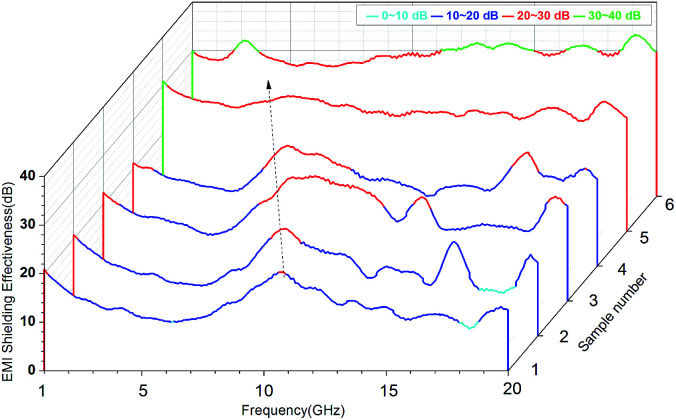
Plots of EMI shielding effectiveness for VGFBTM composites with different loading of fillers in 1.0–20.0 GHz.

EMI SE is a measure of the material's ability to attenuate the electromagnetic wave intensity.^35^ For electromagnetic radiation, EMI SE is the logarithm of the ratio of incident power (*P*_i_) to transmitted power (*P*_t_) in decibels, *i.e.*, SE = 10 log(*P*_i_/*P*_t_). For example, SE of 20 and 30 correspond to the blocking of 99% and 99.9% of electromagnetic incident waves, respectively. Fig. 8 showed EMI SE of the VGFBTM composites sheets of 2.6 mm thickness at room temperature in the range of 1.0–20.0 GHz. In Fig. 8, we found that the SE peaks of the samples move to low frequencies with increasing content of nanofillers, resulting from dielectric loss of the increasing BaTiO_3_ nanoparticles at low frequency.^36,37^ The target value of the EMI SE needed for commercial applications is 20 dB. As presented of the sample no. 1 in Fig. 8, the V1 composites exhibited SE of 20.3 dB at 10.6 GHz with 2.3 wt% GFBTM filler content, indicating the composites can meet the commercial application demands. Besides, the effective EMI SE (SE > 26.7 dB) bandwidth was enlarged from 1.0 to 20.0 GHz with 16.1 wt% GFBTM filler content. In this work, the SE value increased within content of 16.1 wt% GFBTM filler. The excellent SE properties of the nanocomposites attributed to the multiple electromagnetic loss mechanisms, such as magnetic absorption, dielectric absorption, synergistic effect of composite system and the special electromagnetic light effect of nanomaterials.

Theoretically, the relationship between transmittance (*T*), reflectance (*R*), and absorbance (*A*) of a shielding material can be described using eqn (1).

1 *T* + *R* + *A* = 1

The *T* and *R* coefficients were estimated through *S* parameters and related by the following equation:2
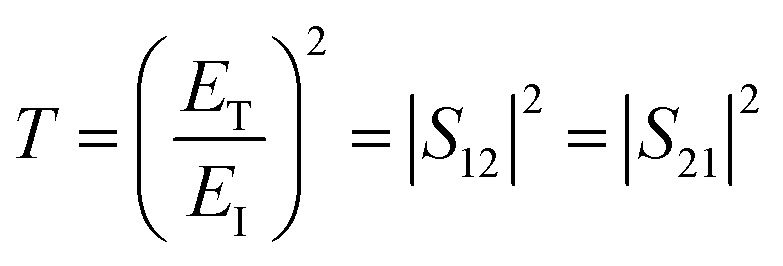
3
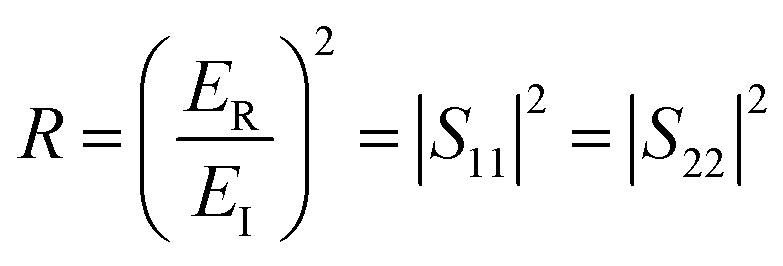


The total EMI SE (SE_total_) is the sum of the absorption (SE_A_), reflection (SE_R_), and multiple reflection (SE_M_) (eqn (4))

4 SE_total_ = SE_A_ + SE_R_ + SE_M_

when SE_total_ > 15 dB, it is usually assumed that (SE_M_ is negligible)

5 SE_total_ ≈ SE_A_ + SE_R_

Hence, the SE_total_ of a shielding material can be written as follow (eqn (6)).6
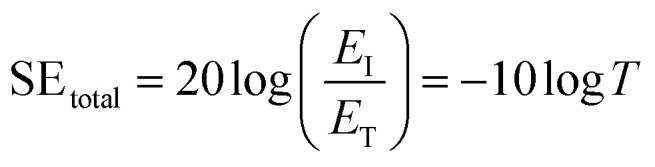


Considering the effective absorbance (*A*_eff_) (eqn (7)), with respect to the power of the incident electromagnetic wave inside the shielding material, the SE_R_ and SE_A_ can be described by eqn (8) and (9).^38^7
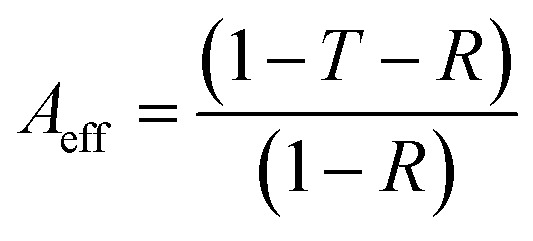

8 SE_R_ = 10 log(1 − R)

9
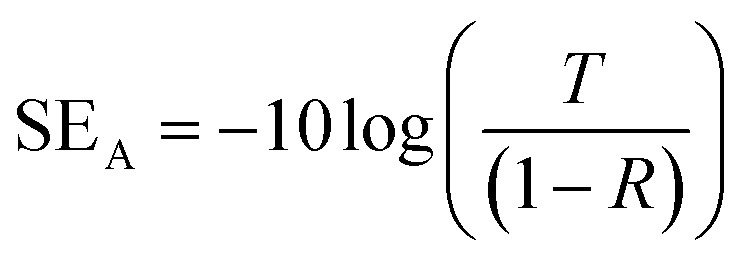


**Fig. 9:**
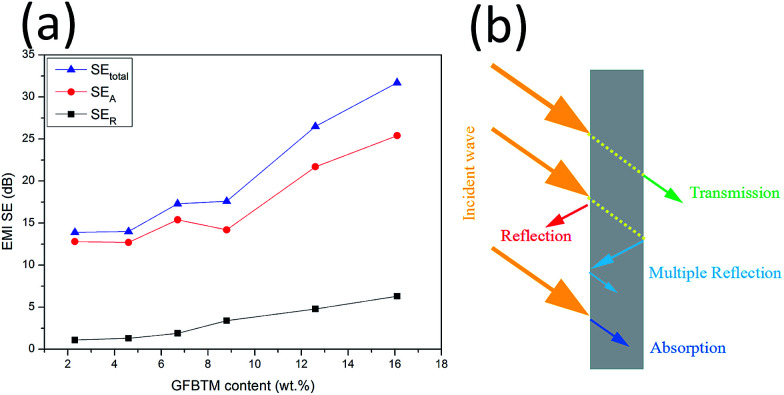
(a) Comparison of SE_total_, SE_A_ and SE_R_ of VGFBTM composites at 3.0 GHz as a function of GFBTM filler content; (b) illustration of the major mechanisms for EMI shielding.

To explore the EMI shielding mechanism, the effects of multi-fillers on SE_total_, SE_A_ and SE_R_ of the VGFBTM composites at 3.0 GHz were calculated and investigated (Fig. 9a). It was evident that SE_A_ > SE_R_ in terms of the VGFBTM composites. Besides, the rate of the increase in microwave absorption was much larger than that of the increase in microwave reflection. For the V6 composite at 3 GHz, the SE_total_, SE_A_, and SE_R_ are 31.7, 25.4, and 6.3 dB, respectively. Therefore, the contribution of the absorbance was 4.0 times larger than that of the reflectance to the total EMI SE. So we could conclude that microwave absorption was the main contributor to the total EMI SE of the VGFBTM composites, meeting with other reports related to the shielding mechanisms of PANI/GN/MWCNTs and PS/GN/Fe_3_O_4_ composites.^39,40^

The good EMI SE could be attributed to several factors. Firstly, GNPs worked as a carrier of FBT nanoparticles that prevented their agglomeration. The formation of GFBT hybrid containing FBT nanoparticles embedded in the GNPs layers enhanced the interfacial polarization of multiphase. Secondly, MWCNTs acted as a bridge to connect GNPs for increasing electrical conductivity and enhancing dielectric loss in electromagnetic field.^34^ Besides, the efficient complementarities between permittivity and permeability which could enhance EMI absorption property.^27,41,42^ In other words, most of the incident microwaves entering the VGFBTM composites were reflected and scattered many times in the multiphase and netty structure building by GFBT hybrid and MWCNTs fillers, and could not escape from the limited space until they were almost absorbed (Fig. 9b). All above described and discussed results intensely support that the VGFBTM composites displayed good EMI SE in a wide frequency range.

## 4. Conclusions

In summary, the VGFBTM composites with high electromagnetic interference shielding were successfully prepared by adding self-prepared GFBT hybrid and MWCNTs in VMQ matrix *via* two-step solvothermal and solution blending methods. The GFBT hybrid was formed by loading Fe_3_O_4_ and BaTiO_3_ nanoparticles on graphene nanoplates step by step through solvothermal method. The hysteresis loops of GFBT hybrid indicated a super paramagnetic feature which meant considerable magnetic loss. With a synergistic effect of GFBT hybrid and MWCNTs, the VGFBTM composites exhibited good electrical conductivity and electromagnetic interference shielding property. In detail, the composite showed greatly broad bandwidth (SE > 26.7 dB) from 1.0 to 20.0 GHz with a 16.1 wt% total filler loading. As a result, the VGFBTM composite possesses high magnetic permeability, dielectric property and good conductivity, making it a novel potential electromagnetic interference shielding materials, such as sheath material for eliminating electromagnetic pollution in wide frequency range.

## Conflicts of interest

There are no conflicts to declare.

## Supplementary Material
